# Prediction model for severe autoimmune encephalitis: a tool for risk assessment and individualized treatment guidance

**DOI:** 10.3389/fneur.2025.1575835

**Published:** 2025-03-18

**Authors:** Zhuxiao Xie, Jingxiao Zhang, Lei Liu, Enyu Hu, Jiawei Wang

**Affiliations:** Department of Neurology, Beijing Tongren Hospital, Capital Medical University, Beijing, China

**Keywords:** autoimmune encephalitis, severe, prediction model, risk stratification, individualized treatment

## Abstract

**Background:**

Severe autoimmune encephalitis (AE) can cause significant neurological deficits, status epilepticus, status dystonicus, and even death, which can be life-threatening to patients. Accurate risk stratification for severe AE progression is critical for optimizing therapeutic strategies. The comprehensive prediction models for severe AE based on routine clinical data and laboratory indicators remain lacking.

**Objective:**

To develop and validate a prediction model for severe AE to optimize individualized treatment.

**Methods:**

We collected clinical data and laboratory examination results from 207 patients with confirmed AE. The study population was divided into development and validation cohort. A prediction model for severe AE was constructed using a nomogram and was rigorously validated both internally and externally. Severe AE was defined as modified Rankin Scale (mRS) > 2 and Clinical Assessment Scale for Encephalitis (CASE) > 4.

**Results:**

The variables ultimately included in the nomogram for the severe AE predictive model were age, psychiatric and/or behavioral abnormalities, seizures, decreased level of consciousness, cognitive impairment, involuntary movements, autonomic dysfunction, and increased intrathecal IgG synthesis rate. It demonstrated excellent discriminative capacity and calibration through internal-external validation.

**Conclusion:**

The prediction model has highly feasibility in clinical practice, and holds promise as an important tool for risk assessment and guiding individualized treatment in patients with AE.

## 1 Introduction

Autoimmune encephalitis (AE) refers to a group of encephalitis mediated by autoimmune mechanisms. It is a rare, potentially disabling, yet treatable condition characterized by heterogeneous clinical manifestations including psychiatric symptoms, cognitive impairment, memory decline, seizures, speech disturbances, motor dysfunction, and alterations in consciousness levels (1). With substantial morbidity and mortality rates that impact patients’ quality of life and impose significant economic burdens on both patients and society (2). AE has garnered increasing attention within the international neurology community. The majority of AE patients respond well to immunotherapy, early diagnosis and timely therapeutic intervention remain critical for optimizing clinical outcomes (3).

Severe AE may can lead to significant neurological deficits, coma, and even death (4). Accurate risk stratification for disease progression is essential for implementing individualized therapeutic strategies. Conventional severity assessment relies on Intensive Care Unit (ICU) admission requirements, modified Rankin Scale (mRS), and the clinical assessment scale for autoimmune encephalitis (CASE) (4–8). Previous studies have investigated risk factors associated with severe AE, however, there were limitations that these studies did not cover all types of AE or could not effectively assess risks at the early stage of disease. The anti-NMDAR Encephalitis One-Year Functional Status (NEOS) score, based on five variables (intensive care unit admission, treatment delay >4 weeks, lack of clinical improvement within 4 weeks, abnormal MRI, and CSF white blood cell (WBC) count >20 cells/μL), can be used to predict the risk of poor functional outcomes at 1 year in patients with anti-NMDAR encephalitis (9). Some other evidences have identified clinical biomarkers including anemia, first-line immunotherapy failure, and elevated CSF interleukin-17a (IL-17a) levels predictors of critical illness progression (3, 10, 11). Nevertheless, in the early stages of hospital admission, there is a lack of comprehensive evaluation indicators for the risk of severe AE. The study integrated clinical manifestations with laboratory test results on admission to construct the first nomogram model for severe AE prediction.

## 2 Materials and methods

### 2.1 Study subjects

**Figure 1:**
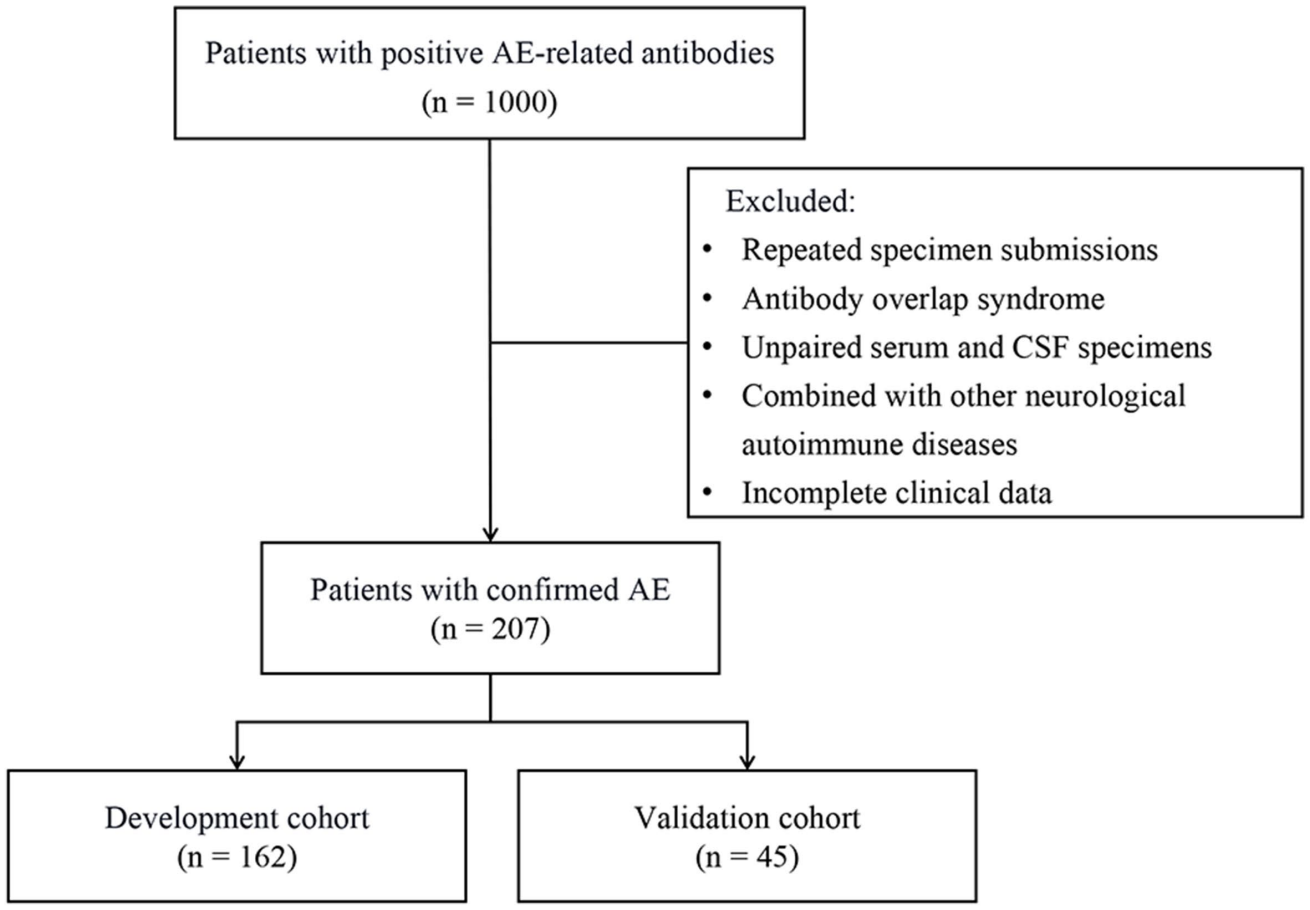
Profile of the inclusion process for study subjects. AE, autoimmune encephalitis; CSF, cerebrospinal fluid.

We collected data from patients suspected of AE who were admitted to our hospital between February 2012 and May 2022. For each patient, paired serum and CSF samples were tested for neuronal antibodies. Study subjects were rigorously selected according to the inclusion and exclusion criteria shown in Table 1 and the enrollment process illustrated in Figure 1. A retrospective analysis was conducted on the clinical profiles and laboratory findings of these subjects. The entire cohort was divided into a development cohort and a validation cohort to construct a prediction model for severe AE, followed by internal and external validations.

**Table 1:** The inclusion and exclusion criteria of study subjects.

Inclusion criteria
Presence of at least one or more of the following clinical features: (1) fever; (2) seizures; (3) memory dicline; (4) cognitive dysfunction; (5) focal CNS neurological deficits; (6) reduced consciousness; (7) psychiatric and/or behavioral abnormalities; (8) sleep disorders; (9) EEG abnormalities; and (10) imaging abnormalities.
Paired detection of serum and CSF samples.
Positive detection of AE-related antibodies in blood and/or CSF using CBA and TBA. TBA methods.
May have tumors associated with AE, such as SELC, teratoma, and thymoma.
Exclusion criteria
Combined with other neurological autoimmune diseases.
Combined with infectious diseases, tuberculous meningitis, etc.
Detection of antibodies in serum only or in cerebrospinal fluid only.
Antibody overlap syndrome.
Incomplete clinical data.
Drug abusers and alcoholics.
Pregnant or lactating women.

AE, autoimmune encephalitis; CSF, cerebrospinal fluid; CBA, cell-based assay; EEG, electroencephalogram; TBA, tissue-based assay; SELC, small cell lung cancer.

### 2.2 Neuronal antibodies detection

Using the cell-based assay (CBA) method: (1) Commercial CBA kits (FA112d-1005-1, Euroimmun AG, Lübeck, Germany; MT226-16 and MT29916, Pulse Biotechnology Co., Ltd., Shaanxi, China) were utilized to detect AE antibodies in both serum and CSF of patients, including anti-N-methyl-D-aspartate receptor (NMDAR) antibody, anti-*α*-amino-3-hydroxy-5-methyl-4-isoxazolepropionic acid receptor (AMPAR) antibody, anti-dipeptidyl-peptidase-like protein 6 (DPPX) antibody, anti-gamma-aminobutyric acid-B receptor (GABA_B_R) antibody, anti-leucine-rich glioma-inactivated protein 1 (LGI1) antibody, anti-contactin-associated protein-like 2 (CASPR2) antibody, anti-metabotropic glutamate receptor 5 (mGluR5) antibody, anti-IgLON5 antibody, anti-glial fibrillary acidic protein (GFAP) antibody and anti-glutamic acid decarboxylase 65 (GAD65) antibody. The initial dilution concentration for CSF was 1:1, and for serum, it was 1:10, according to the manufacturers’ instructions.

### 2.3 Collection of clinical data and laboratory test results

The Case Report Form (CRF) was used to collect medical record information of the study subjects, which mainly included the following contents: Demographic information: including name, gender, and age. Neuronal antibodies detection results: including types of antibodies and their titers. Clinical manifestations: including the presence of prodromal infection symptoms (such as upper respiratory tract infection symptoms, headache, fever, diarrhea, etc.) and whether there was a concurrent tumor. CSF laboratory tests: including intracranial pressure, CSF cell count, CSF protein quantification, and intrathecal IgG synthesis rate. Imaging results: whether cranial magnetic resonance imaging (MRI) showed abnormalities. Electrophysiological examination: whether electroencephalogram (EEG) results were abnormal. Assessment of disease severity at peak: evaluated using the mRS and the CSAE score.

### 2.4 Related definitions of the study

Antibody titers: A titer of 1:320 was defined as strongly positive, a titer of 1:100 was defined as positive, and titers of 1:32 and 1:10 were defined as weakly positive. Abnormal cranial MRI: Abnormalities on cranial MRI included high signal lesions on T2-weighted fluid-attenuated inversion recovery (T2 Flair) sequences, highly localized to one or both medial temporal lobes (limbic encephalitis), or multiple lesions involving the gray matter, white matter, or both. Abnormal EEG: EEG findings indicating any of the following conditions including abnormal state changes, focal or diffuse slow waves, slow wave rhythms, epileptiform discharges, or extreme δ brushes. Severe AE: It was defined as mRS > 2 and CASE >4 (8, 12, 13).

### 2.5 Statistical analysis

Statistical analysis was performed using SPSS 26.0 software. Categorical variables were described using composition ratios and frequencies, with inter-group comparisons conducted using the χ^2^ test or Fisher’s exact test. For continuous variables, normality tests were performed. Normally distributed data were described using the mean ± standard deviation ( $\bar{x}\pm s$ ), with inter-group comparisons conducted using independent samples t-tests or ANOVA, and correlation analyses performed using Pearson correlation. Non-normally distributed data were described using the median (minimum, maximum) [M (range)], with inter-group analyses conducted using the Mann–Whitney U test, and correlation analyses performed using Spearman correlation. Binary logistic regression was used for regression analysis of dichotomous variables. A *p* value <0.05 was considered statistically significant. Model construction (nomogram) and internal validation using the Bootstrap method were performed using R software (R version 4.3.0). The following packages were utilized: “rms,” “Hmisc,” “boot,” and “nomogramFormula.”

## 3 Results

### 3.1 Baseline characteristics of the study subjects

**Figure 2:**
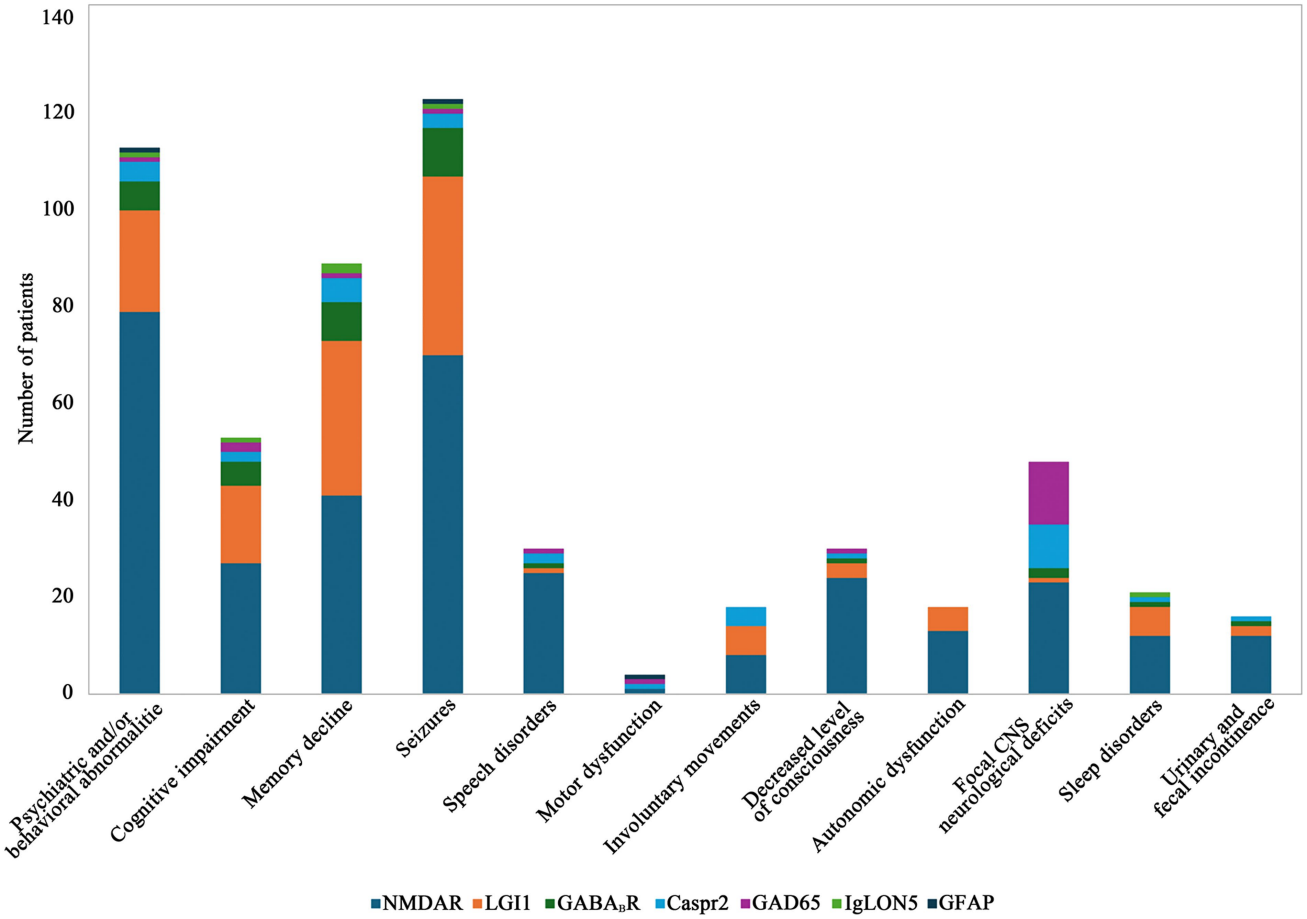
Initial clinical manifestations of AE. NMDAR, N-methyl-D-aspartate receptor; AMPAR, anti-α-amino-3-hydroxy-5-methyl-4-isoxazolepropionic acid receptor; DPPX, dipeptidyl-peptidase-like protein 6; GABA_B_R, gamma-aminobutyric acid-B receptor; LGI1, leucine-rich glioma-inactivated protein 1; CASPR2, contactin-associated protein-like 2; mGluR5, metabotropic glutamate receptor 5; GFAP, glial fibrillary acidic protein; GAD65, glutamic acid decarboxylase 65; CNS, central nervous system.

Initial screening identified 1,000 patients with AE antibodies positivity. After excluding cases with antibody overlap syndrome, unpaired serum and CSF specimens, coexistence of other immune diseases, and incomplete clinical information, a final cohort of 207 patients with confirmed AE was included in this study, which comprised of 109 cases of anti-NMDAR encephalitis, 48 cases of anti-LGI1 encephalitis, 14 cases of anti-GABA_B_R encephalitis, 16 cases of anti-Caspr2 encephalitis, 16 cases of anti-GAD65 encephalitis, and 2 cases each of anti-IgLON5 encephalitis and anti-GFAP encephalitis. Among these patients, 124 were male (58.6%) and 83 were female (41.4%), with no significant intergroup gender distribution differences across antibody-specific subgroups (*χ*^2^ = 1.882, *p* value = 0.757). Comprehensive clinical data encompassing demographics, laboratory parameters (including CSF-WBC, CSF-Pro, intrathecal IgG synthesis rates), neuroimaging (abnormal cranial MRI), neurophysiological findings (abnormal EEG), immunotherapy responsiveness, and paraneoplastic associations are detailed in Table 2. (Note: Anti-IgLON5 and anti-GFAP encephalitis cases were excluded from tabular presentation due to limited sample size [*n* = 2 each]). Prodromal manifestations occurred in 67 patients (32.4%), presented with prodromal symptoms, primarily including headache, fever, upper respiratory tract infection, and diarrhea. The initial clinical manifestations were most commonly seizures (37.68%), followed by psychiatric and/or behavioral abnormalities (20.77%) and memory decline (13.53%) (Figure 2).

**Table 2:** Baseline characteristics of the study subjects.

	Total (*n* = 207)	NMDAR (*n* = 109)	LGI1 (*n* = 48)	GABABR (*n* = 14)	Caspr2 (*n* = 16)	GAD65 (*n* = 16)	*p* vaule
Gender, Male	124 (58.6)	67 (61.5)	27 (56.3)	10 (71.4)	9 (56.3)	8 (50.0)	0.757
Age	41 (11–82)	33 (11–17)	57 ± 11^*^	61 ± 14^*^	43 ± 16^*^	53 ± 13^*^	**<0.001**
Prodromal infection	67 (32.4)	49 (45.0)	7 (14.6)	2 (14.3)	5 (31.3)	4 (25.0)	**0.001**
Psychiatric and/or behavioral abnormalities	113 (54.6)	79 (72.5)	21 (43.8)	6 (42.9)	4 (25.0)	1 (6.3)	**<0.001**
Cognitive impairment	53 (25.6)	27 (24.8)	16 (33.3)	5 (35.7)	2 (12.5)	2 (12.5)	0.287
Memory decline	89 (43.0)	41 (37.6)	32 (66.7)	8 (57.1)	5 (31.3)	1 (6.3)	**<0.001**
Seizures	123 (59.4)	70 (64.2)	37 (77.1)	10 (71.4)	3 (18.8)	1 (6.3)	**<0.001**
Speech disorders	30 (14.5)	25 (22.9)	1 (2.1)	1 (7.1)	2 (12.5)	1 (6.3)	**0.005**
Motor dysfunction	4 (1.9)	1 (0.9)	0	0	1 (6.3)	1 (6.3)	0.180
Involuntary movement	18 (8.7)	8 (7.3)	6 (12.5)	0	4 (25.0)	0	0.079
Decreased level of consciousness	30 (14.5)	24 (22.0)	3 (6.3)	1 (7.1)	1 (6.3)	1 (6.3)	0.056
Autonomic dysfunction	31 (15.0)	22 (20.2)	7 (14.6)	1 (7.1)	1 (6.3)	0	0.246
Focal CNS neurological deficits	48 (23.2)	23 (21.1)	1 (2.1)	2 (14.3)	9 (56.3)	13 (81.3)	**<0.001**
Urinary and bowel dysfunction	16 (7.7)	12 (11.0)	2 (4.2)	1 (7.1)	1 (6.3)	0	0.512
ICU Admission	22 (10.6)	18 (16.5)	1 (2.1)	1 (7.1)	1 (6.3)	1 (6.3)	0.066
Increased ICP	50 (24.2)	33 (30.3)	8 (16.7)	4 (28.6)	1 (6.3)	3 (18.8)	0.144
Increased CSF-WBC	107 (51.7)	66 (60.6)	19 (39.6)	8 (57.1)	9 (56.3)	4 (25.0)	**0.024**
Increased CSF-Pro	71 (34.3)	37 (33.9)	14 (29.2)	8 (57.1)	5 (31.3)	6 (37.5)	0.431
Increased intrathecal IgG synthesis rate	51 (24.7)	28 (25.7)	9 (18.8)	5 (35.7)	5 (31.3)	4 (25.0)	0.654
Abnormal cranial MRI	129 (62.4)	70 (64.2)	30 (62.5)	11 (78.5)	10 (62.5)	6 (37.5)	0.203
Abnormal EEG	166 (80.2)	86 (78.9)	38 (79.2)	12 (85.7)	14 (87.5)	14 (87.5)	0.906
Associated with tumors	18 (8.7)	5 (4.6)	2 (4.2)	8 (57.1)	1 (6.3)	2 (12.5)	**<0.001**

* For multiple group comparisons, a corrected *p* value was applied, with *p* value < 0.01 considered statistically. The bold values indicated statistically significant results.

NMDAR, N-methyl-D-aspartate receptor; GABA_B_R, gamma-aminobutyric acid-B receptor; LGI1, leucine-rich glioma-inactivated protein 1; CASPR2, contactin-associated protein-like 2; GAD65, glutamic acid decarboxylase 65; CNS, central nervous system; CSF, cerebrospinal fluid; CSF-WBC, cerebrospinal fluid white blood cell; CSF-Pro, cerebrospinal fluid protein; EEG, electroencephalogram; ICU, Intensive Care Unit; ICP, intracranial pressure; MRI, magnetic resonance imaging.

### 3.2 Assessment of factors influencing disease severity

The study population was divided into a development cohort comprising 162 cases and a validation cohort consisting of 45 cases (Table 3). This division facilitated the construction of a prediction model for severe AE followed by rigorous validation protocols.

**Table 3:** Comparison of data between development and validation cohorts.

	Development cohort (*n* = 162)	Validation cohort (*n* = 45)	*p* value
Gender, Male	98 (60.5)	26 (57.8)	0.864
Age	43 (11–82)	44 (13–77)	0.671
Prodromal infection	50 (30.9)	17 (37.8)	0.471
Clinical manifestation			
Behavioral and psychiatric abnormalities	94 (58.0)	19 (42.2)	0.065
Cognitive impairment	45 (27.8)	8 (17.8)	0.185
Memory decline	71 (43.8)	18 (40.0)	0.734
Epileptic seizures	102 (63.0)	21 (46.7)	0.059
Speech disorders	23 (14.2)	7 (15.6)	0.819
Motor dysfunction	3 (1.9)	1 (2.20)	0.873
Involuntary movement	13 (8.0)	5 (11.1)	0.551
Decreased level of consciousness	26 (16.0)	4 (8.9)	0.227
Dysautonomia	21 (13.0)	10 (22.2)	0.155
Focal CNS damage	31 (19.1)	17 (37.8)	0.011
Sleep disorders	14 (8.6)	7 (15.6)	0.261
ICU admission	17 (10.5)	5 (11.1)	0.905
Increased ICP	41 (25.3)	9 (20.0)	0.557
Increased CSF-WBC	92 (56.8)	15 (33.3)	0.005
Increased CSF-Pro	56 (34.6)	15 (33.3)	0.877
Increased intrathecal IgG synthesis rate	41 (25.3)	10 (22.2)	0.702
Abnormal cranial MRI	98 (60.5)	31 (68.9)	0.358
Abnormal EEG	130 (80.2)	36 (80.0)	0.971
Strongly positive serum AE antibodies	32 (19.8)	4 (8.9)	0.119
Strongly positive CSF AE antibodies	70 (43.2)	6 (13.3)	<0.001
Associated with tumors	17 (10.5)	1 (2.20)	0.131

AE, autoimmune encephalitis; CNS, central nervous system; CSF, cerebrospinal fluid; CSF-WBC, cerebrospinal fluid white blood cell; CSF-Pro, cerebrospinal fluid protein; EEG, electroencephalogram; ICU, Intensive Care Unit; ICP, intracranial pressure; MRI, magnetic resonance imaging.

#### 3.2.1 Analysis of factors influencing disease severity based on mRS

Initial univariate analysis of the development cohort (mRS > 2 stratification) identified age (*p* value = 0.023), psychiatric and/or behavioral abnormalities (*p* value = 0.002), cognitive impairment (*p* value = 0.002), autonomic dysfunction (*p* value = 0.005), and CSF antibody titers (*p* value = 0.017) as severity-associated factors (Table 4). Multivariate logistic regression (variables with *p* value < 0.1) demonstrated significant associations with: age: OR 1.03 (95% CI 1.00 - 1.05, *p* value = 0.021), psychiatric and/or behavioral abnormalities: OR 3.37 (95% CI 1.56 - 7.27, *p* value = 0.002), cognitive impairment OR 3.36 (95% CI 1.24 - 9.12, *p* value = 0.017), and autonomic dysfunction: OR 8.89 (95% CI 1.11 - 71.54, *p* value = 0.040) (Table 5).

**Table 4:** Univariate analysis of factors associated with severe AE (based on mRS).

	mRS ≤ 2 (*n* = 52)	mRS > 2 (*n* = 110)	*p* value
Gender, Male	67 (60.9)	31 (59.6)	>0.999
Age	38 (12–80)	45 (11–82)	**0.023**
Prodromal infection	12 (23.1)	38 (34.5)	0.150
Clinical manifestation			
Psychiatric and/or behavioral abnormalities	21 (40.4)	73 (66.4)	**0.002**
Cognitive impairment	6 (11.5)	39 (35.5)	**0.002**
Memory decline	25 (48.1)	46 (41.8)	0.500
Seizures	33 (63.5)	69 (62.7)	>0.999
Speech disorders	5 (9.6)	18 (16.4)	0.337
Motor dysfunction	1 (1.9)	2 (1.8)	>0.999
Involuntary movement	3 (5.8)	10 (9.1)	0.552
Decreased level of consciousness	4 (7.7)	22 (20.0)	0.065
Autonomic dysfunction	1 (1.9)	20 (18.2)	**0.005**
Focal CNS neurological deficits	7 (13.5)	24 (21.8)	0.285
Sleep disorders	1 (1.9)	13 (11.8)	0.068
Increased ICP	16 (30.8)	25 (22.7)	0.333
Increased CSF-WBC	28 (53.8)	64 (58.2)	0.615
Increased CSF-Pro	12 (23.1)	44 (40.0)	0.051
Increased intrathecal IgG synthesis rate	8 (15.4)	33 (30.0)	0.054
Abnormal cranial MRI	28 (53.8)	70 (63.6)	0.302
Abnormal EEG	43 (82.7)	87 (79.1)	0.676
Strongly positive serum AE antibodies	6 (11.5)	26 (23.6)	0.091
Strongly positive CSF AE antibodies	15 (28.8)	55 (50.0)	**0.017**
Associated with tumors	2 (3.8)	15 (13.6)	0.096

The bold values indicated statistically significant results. AE, autoimmune encephalitis; CNS, central nervous system; CSF, cerebrospinal fluid; CSF-WBC, cerebrospinal fluid white blood cell; CSF-Pro, cerebrospinal fluid protein; EEG, electroencephalogram; ICP, intracranial pressure; MRI, magnetic resonance imaging; mRS, modified Rankin Scale.

**Table 5:** Multivariate analysis of factors associated with severe AE (based on mRS).

	OR (95% CI)	*p* value
Age	1.02 (1.00–1.05)	0.021
Psychiatric and/or behavioral abnormalities	3.37 (1.56–7.27)	0.002
Cognitive impairment	3.36 (1.24–9.12)	0.017
Autonomic dysfunction	8.89 (1.11–71.54)	0.040

AE, autoimmune encephalitis; mRS, modified Rankin Scale.

#### 3.2.2 Analysis of factors influencing disease severity based on CASE

Subsequent analysis using CASE >4 stratification revealed additional risk factors: psychiatric and/or behavioral abnormalities (*p* value <0.001), seizures (*p* value = 0.004), decreased consciousness level (*p* value <0.001), autonomic dysfunction (*p* value = 0.007), increased intrathecal IgG synthesis rate (*p* value = 0.025), and strong positivity for AE antibodies in CSF (*p* value = 0.003) (Table 6).

**Table 6:** Univariate analysis of factors associated with severe AE (based on CASE).

	CASE ≤4 (*n* = 100)	CASE >4 (*n* = 62)	*p* value
Gender, Male	58 (58.0%)	40 (64.5%)	0.509
Age	40.5 (12–82)	45 (11–80)	0.581
Prodromal infection	35 (35.0%)	15 (24.2%)	0.165
Clinical manifestation			
Psychiatric and/or behavioral abnormalities	46 (46.0%)	48 (77.4%)	**<0.001**
Cognitive impairment	23 (23.0%)	22 (35.5%)	0.105
Memory decline	42 (42.0%)	29 (46.8%)	0.626
Seizures	54 (54.0%)	48 (77.4%)	**0.004**
Speech disorders	11 (11.0%)	12 (19.4%)	0.167
Motor dysfunction	1 (1.0%)	2 (3.2%)	0.559
Involuntary movement	5 (5.0%)	8 (12.9%)	0.082
Decreased level of consciousness	7 (7.0%)	19 (30.6%)	**<0.001**
Autonomic dysfunction	7 (7.0%)	14 (22.6%)	**0.007**
Focal CNS neurological deficits	19 (19.0%)	12 (19.4%)	>0.999
Sleep disorders	6 (6.0%)	8 (12.9%)	0.155
Increased ICP	24 (24.0%)	17 (27.4%)	0.711
Increased CSF-WBC	53 (53.0%)	39 (62.9%)	0.255
Increased CSF-Pro	33 (33.0%)	23 (37.1%)	0.614
Increased intrathecal IgG synthesis rate	19 (19.0%)	22 (35.5%)	**0.025**
Abnormal cranial MRI	58 (58.0%)	40 (64.5%)	0.509
Abnormal EEG	80 (80.0%)	50 (80.6%)	>0.999
Strongly positive serum AE antibodies	17 (17.0%)	15 (24.2%)	0.312
Strongly positive CSF AE antibodies	34 (34.0%)	36 (58.1%)	**0.003**
Associated with tumors	9 (9.0%)	8 (12.9%)	0.599

The bold values indicated statistically significant results. AE, autoimmune encephalitis; CASE, clinical assessment scale for autoimmune encephalitis; CNS, central nervous system; CSF, cerebrospinal fluid; CSF-WBC, cerebrospinal fluid white blood cell; CSF-Pro, cerebrospinal fluid protein; EEG, electroencephalogram; ICP, intracranial pressure; MRI, magnetic resonance imaging.

Variables with *p* value <0.1 were included in the multivariate logistic regression analysis. The results showed that psychiatric and/or behavioral abnormalities OR 3.70 (95% CI 1.67–8.16, *p* value = 0.001), seizures OR 6.07 (95% CI 2.42–15.22, *p* value <0.001), involuntary movement OR 4.23 (95% CI 1.00–17.84, *p* value = 0.049), decreased level of consciousness OR 6.80 (95% CI 2.18–21.21, *p* value = 0.001), and increased intrathecal IgG synthesis rate OR 3.05 (95% CI 1.23–7.50, *p* value = 0.015) were associated with the severity of AE (Table 7).

**Table 7:** Multivariate analysis of severe AE (based on CASE).

	OR (95% CI)	*p* value
Psychiatric and/or behavioral abnormalities	3.70 (1.67–8.16)	0.001
Seizures	6.07 (2.42–15.22)	<0.001
Involuntary movement	4.23 (1.00–17.84)	0.049
Decreased level of consciousness	6.80 (2.18–21.21)	0.001
Increased intrathecal IgG synthesis rates	3.05 (1.23–7.50)	0.015

AE, autoimmune encephalitis; CASE, clinical assessment scale for autoimmune encephalitis.

### 3.3 Severe AE prediction model

#### 3.3.1 Construction of a severe AE prediction model

**Figure 3:**
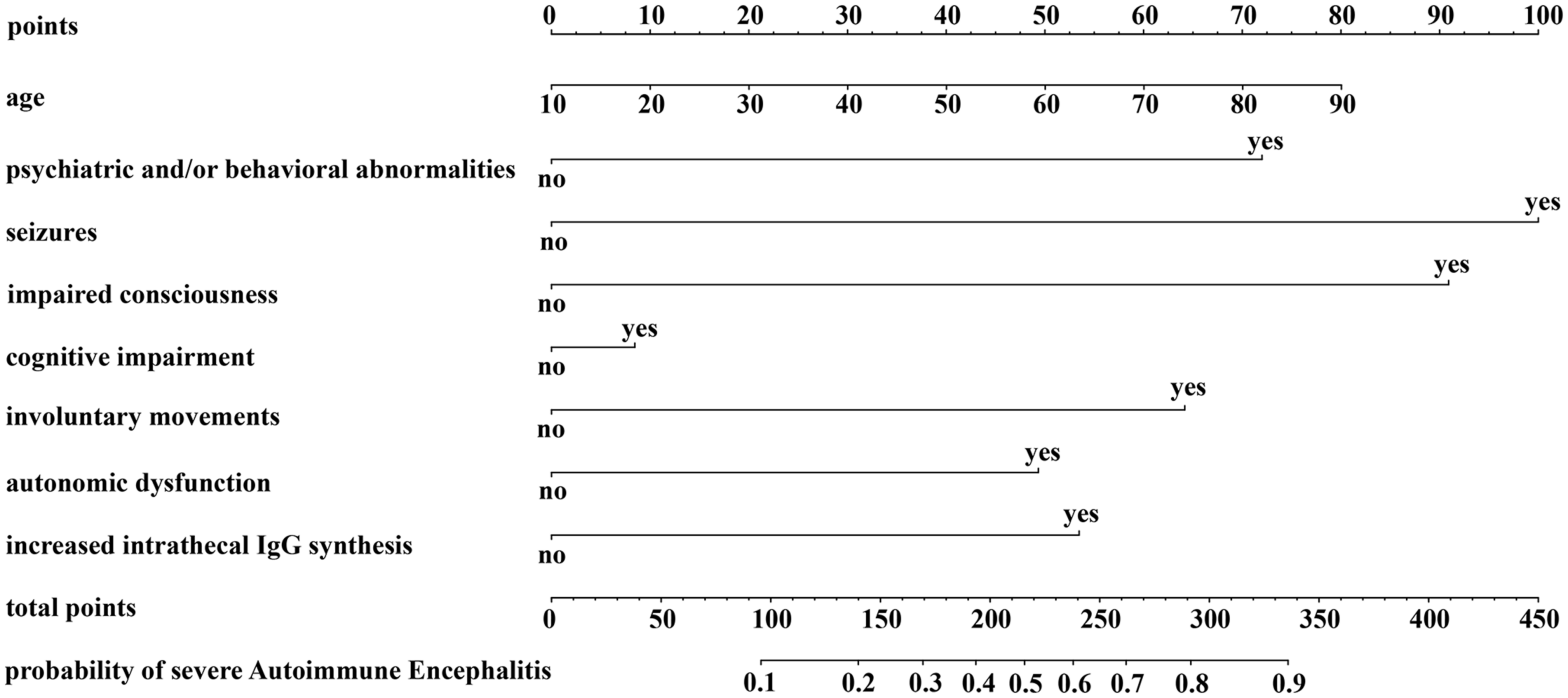
Prediction model for severe AE (nomogram).

In this study, severe AE was defined as an mRS > 2 and CASE >4. A nomogram method was employed to construct a predictive model for severe AE by incorporating variables with statistical significance (*p* value <0.05) from the multivariate analysis. The variables included age, psychiatric and/or behavioral abnormalities, seizures, decreased level of consciousness, cognitive impairment, involuntary movements, autonomic dysfunction, and increased intrathecal IgG synthesis rate. As shown in Figure 3, vertical lines drawn on the scale lines representing each influencing factor yield individual scores for each factor. The total score is obtained by summing the scores of the eight influencing factors. A vertical line plotted on the final axis corresponds to the predicted probability of severe AE.

#### 3.3.2 The internal validation of the severe AE prediction model

**Figure 4:**
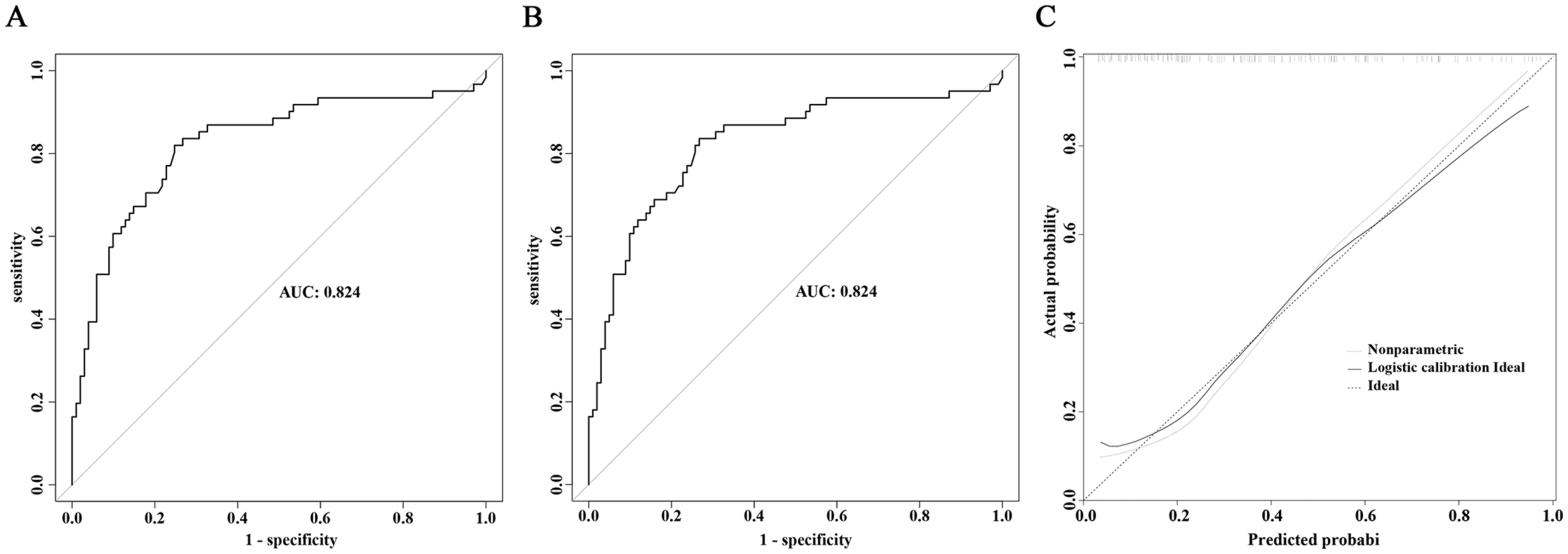
ROC and calibration curves of the severe AE prediction model. **(A)** ROC curve of the prediction model in the development cohort. **(B)** ROC curve of the prediction model after internal validation using the Bootstrap method. **(C)** Calibration curve of the prediction model in the development cohort.

The efficacy of the prediction model was determined by plotting the ROC curve analysis. In the development cohort, the area under the curve (AUC) was 0.824 (95% CI: 0.752–0.896), with a sensitivity of 0.820 and specificity of 0.752, *p* vaule <0.001 (Figure 4A). For internal validation using the Bootstrap method, after performing 1,000 resamples, the ROC curve was plotted again, the AUC was 0.824 (95% CI: 0.752–0.896), with a sensitivity of 0.836 and specificity of 0.733, *p* value <0.001 (Figure 4B). The nomogram prediction model was calibrated by comparing the predicted probabilities of severe AE with the actual diagnosis probabilities after bias correction. There was good consistency between the predicted and actual probabilities, indicating that the prediction model was well-calibrated (Figure 4C).

#### 3.3.3 External validation of the severe AE prediction model

**Figure 5:**
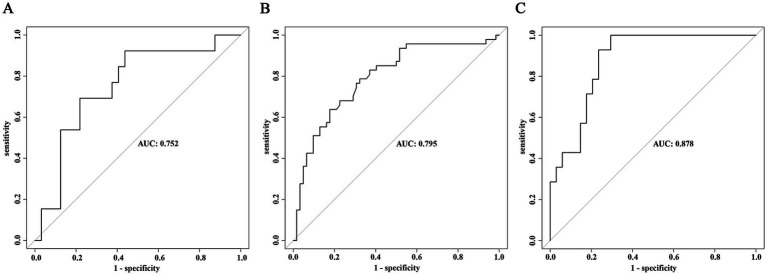
External validation of the prediction model for severe AE. **(A)** ROC curve of the predictive model in the overall validation cohort. **(B)** ROC curve of the predictive model in the validation cohort for anti-NMDAR encephalitis. **(C)** ROC curve of the predictive model in the validation cohort for anti-LGI1 encephalitis.

The external validation of the model was divided into three parts. First, external validation was performed using 45 patients from the validation cohort, resulting in an AUC of 0.756 (95% CI 0.593–0.912), *p* value = 0.002 (Figure 5A). Additionally, to evaluate the model’s predictive efficacy for severe AE associated with different antibody types, external validation was conducted separately on the 109 cases of anti-NMDAR encephalitis and 48 cases of anti-LGI1 encephalitis included in this study. The results showed that for the anti-NMDAR antibody encephalitis cohort, the AUC was 0.795 (95% CI 0.709–0.881), *p* value <0.001 (Figure 5B), and for the anti-LGI1 encephalitis cohort, the AUC was 0.878 (95% CI 0.784–0.972), *p* value <0.001 (Figure 5C).

## 4 Discussion

AE represents a rare neuroimmunological disorder characterized by acute to subacute neuropsychiatric manifestations and significant disability rates (2). Recent advancements in autoantibody detection techniques have facilitated substantial progress in AE research. While immunotherapy demonstrates efficacy in many cases, 11.2–55.0% of patients progress to severe disease (14–16), often complicated by respiratory failure requiring mechanical ventilation (17), hemodynamic instability caused by severe autonomic dysfunction (18, 19), status dystonicus (SD) (20), coma, and status epilepticus (SE) (4, 21), which can be life-threatening to patients. Early intervention (within 8 days) with combined steroid pulse therapy and intravenous immunoglobulin (IVIG) has been established as an independent predictor of favorable outcomes, while antibody testing for various causes of encephalopathy need to be prior to initiation of immunotherapy (22–24). Accurate risk stratification for severe AE progression is therefore critical for optimizing therapeutic strategies. Despite some studies on disease progression prediction factors in AE (25–27), a systematic, large-scale data-based study to construct a prediction model for severe AE in the early stages of patient admission remains lacking. Our study retrospectively analyzed the clinical data and laboratory examination results of 207 AE cases, constructed a comprehensive prediction model for severe AE, demonstrating excellent discriminative capacity and calibration through rigorous validation. The included variables comprised psychiatric and/or behavioral abnormalities, seizures, decreased level of consciousness, cognitive impairment, involuntary movements, associated with tumors, and increased intrathecal IgG synthesis rate. The mRS score focuses on the overall functional status of patients but lacks the assessment of non-motor symptoms, while the CASE score addresses this limitation, however, its incorporation of laboratory indicators is insufficient. Therefore, in our study, the assessment of disease severity integrated both of the scales, avoiding the bias associated with the use of a single scoring system, which is one of the strengths of our model. Moreover, this is the first nomogram prediction model for severe AE, constructed based on indicators obtained in the early stages of patient admission, thereby ensuring high timeliness. Previous studies have predominantly focused on indicators such as ICU admission, delayed treatment beyond 4 weeks, no clinical improvement within 4 weeks and so on, which were mostly related to the treatment phases. Our model is capable of predicting the risk of severe disease in AE patients at the early admission stage, providing a significant advantage for early intervention and management. The prediction model is expected to be used for future individualized treatment guidance.

The analysis revealed that age was significantly associated with disease severity, consistent with previous reports indicating poorer short-term outcomes in elderly patients (28). This association may be attributed to the higher prevalence of comorbidities in older individuals, as well as the advanced average age of patients with anti-GABA_B_R encephalitis and anti-LGI1 encephalitis. Notably, anti-GABA_B_R encephalitis is frequently associated with tumors, contributing to its relatively worse prognosis. Cognitive impairment was also identified as a significant factor influencing disease severity, aligning with prior studies (29, 30). This may be explained by the fact that patients with cognitive impairment often require increased caregiving support, leading to higher mRS scores. Approximately 50% of severe AE patients exhibited autonomic dysfunction (17), with urinary incontinence/retention being the primary manifestation of autonomic or central nervous system injury. A study of 70 AE patients similarly highlighted urinary incontinence as a predictor of poor prognosis (31), and our findings further confirmed a strong correlation between autonomic dysfunction and AE severity. Additionally, ICU admission was an independent risk factor for poor disease prognosis (9, 32).

In this study, the univariate analysis of the correlation between AE antibody titers and disease severity found that CSF antibody titers were associated with disease severity, whereas serum antibody titers showed no such correlation. However, logistic regression analysis did not identify a statistically significant relationship between antibody titers and disease severity. Consistent with multiple international studies, the association between AE antibody titers and disease severity remains controversial (33–35). A previous study demonstrated that CSF antibody titers were positively correlated with ICU admission, ventilator use, and the presence of concurrent tumors. Furthermore, the study suggested that these three indicators could reflect disease severity. However, no direct correlation was found between CSF antibody titers and prognosis (35). The continuous intrathecal synthesis of neuronal antibodies does not necessarily indicate that encephalitis is in an active phase, and the antibodies titers cannot directly reflect the severity of the condition. Moreover, in CSF analysis, it has been observed that the intrathecal IgG synthesis rate may correlate with the severity of the disease. Although CSF-protein levels and CSF-WBC counts show no statistical significance, previous studies suggest that these parameters often increase within a few days after the onset of neurological symptoms (36). Therefore, monitoring the dynamic changes in CSF components could provide meaningful insights into the progression of the disease.

SE represents a common severe AE manifestation (4, 37, 38), and conventional antiepileptic drugs available in clinical practice are ineffective for approximately one-third of epilepsy patients. The combination of ketogenic diet and stiripentol appeared to constitute effective treatment in SE caused by anti-NMDAR encephalitis (39). SE often accompanied by characteristic EEG abnormalities such as excessive beta activity and extreme delta brush (40). Therefore, EEG monitoring is highly necessary. Although our study did not find a correlation between SE and disease severity, we consider this result to be due to the lack of distinction in seizure types and the degree of EEG abnormalities in our analysis. We recommend long-term EEG monitoring to promptly detect the occurrence of epilepsy. Similarly, no significant correlation was found between cranial MRI abnormalities and disease severity in our study. The absence of cranial MRI-severity correlation in our study contrasts with pediatric cohort findings (41), but aligns with adult studies (29, 42, 43). Furthermore, regarding the tumors associated with AE, anti-NMDAR encephalitis is most commonly associated with ovarian teratomas, while anti-AMPAR encephalitis may be related to thymomas, small cell lung cancer (SCLC), and breast cancer (44). Approximately 50% of patients with anti-GABA_B_R encephalitis are associated with SCLC, which is an important factor for poor prognosis (45, 46). While anti-LGI1 and anti-CASPR2 antibody encephalitis may be associated with thymoma (47, 48). Our study found an association between the presence of tumors and high mRS scores. There has a previous case report of anti-NMDAR encephalitis with bilateral ovarian teratomas supported this conclusion. The patient’s clinical condition rapidly improved following total enucleation of the bilateral ovaries (49). This highlights the importance of comprehensive tumor screening and early surgical intervention when feasible in the treatment of AE.

This study introduces the first nomogram-based prediction model for severe AE, demonstrating excellent discriminative capacity and calibration through rigorous validation. The model’s clinical utility is enhanced by its reliance on readily available parameters. However, several limitations warrant consideration. The single-center retrospective design limited the rare AE subtype representation and ethnic homogeneity (Chinese cohort), which needs more diverse study populations to further validate the generalizability of the conclusions.

## 5 Conclusion

This study has constructed a comprehensive prediction model for severe AE using a variety of readily available clinical indicators. Following rigorous internal-external validation, this model demonstrated excellent performance, enabling personalized risk stratification and therapeutic optimization for AE patients.

## Data Availability

The original contributions presented in the study are included in the article/supplementary material, further inquiries can be directed to the corresponding author.
